# Evaluation of Hemagglutination Activity of Chitosan Nanoparticles Using Human Erythrocytes

**DOI:** 10.1155/2015/247965

**Published:** 2015-02-11

**Authors:** Jefferson Muniz de Lima, Ronaldo Rodrigues Sarmento, Joelma Rodrigues de Souza, Fábio André Brayner, Ana Paula Sampaio Feitosa, Rafael Padilha, Luiz Carlos Alves, Isaque Jerônimo Porto, Roberta Ferreti Bonan Dantas Batista, Juliano Elvis de Oliveira, Eliton Souto de Medeiros, Paulo Rogério Ferreti Bonan, Lúcio Roberto Castellano

**Affiliations:** ^1^7 Grupo de Estudos e Pesquisas em Imunologia Humana (GEPIH), Escola Tecnica de Saúde da UFPB, Universidade Federal da Paraíba, 58051-900 João Pessoa, PB, Brazil; ^2^Departamento de Fisiologia e Patologia, Centro de Ciencias da Saúde, Universidade Federal da Paraíba, 58051-900 João Pessoa, PB, Brazil; ^3^Centro de Pesquisas Aggeu Magalhães (CPqAM/FIOCRUZ) e Laboratório de Imunopatologia Keio Asami (LIKA), Universidade Federal de Pernambuco, 50670-901 Recife, PE, Brazil; ^4^Departamento de Engenharia de Materiais, Centro de Tecnologia, Universidade Federal da Paraíba, 58051-900 João Pessoa, PB, Brazil; ^5^Núcleo de Biomateriais, NEPBIO, Centro de Ciências da Saúde, Universidade Federal da Paraíba, 58051-900 João Pessoa, PB, Brazil; ^6^Departamento de Engenharia, Universidade Federal de Lavras, 37200-000 Lavras, MG, Brazil; ^7^Departamento de Odontologia Clínica e Social, Centro de Ciencias da Saúde, Universidade Federal da Paraíba, 58051-900 João Pessoa, PB, Brazil

## Abstract

Chitosan is a polysaccharide composed of randomly distributed chains of *β*-(1-4) D-glucosamine and N-acetyl-D-glucosamine. This compound is obtained by partial or total deacetylation of chitin in acidic solution. The chitosan-based hemostatic agents have been gaining much attention in the management of bleeding. The aim of this study was to evaluate in vitro hemagglutination activity of chitosan nanoparticles using human erythrocytes. The preparation of nanoparticles was achieved by ionotropic gelification technique followed by neutralization with NaOH 1 mol/L^−1^. The hemagglutination activity was performed on a solution of 2% erythrocytes (pH 7.4 on PBS) collected from five healthy volunteers. The hemolysis determination was made by spectrophotometric analysis. Chitosan nanoparticle solutions without NaOH addition changed the reddish colour of the wells into brown, suggesting an oxidative reaction of hemoglobin and possible cell lysis. All neutralized solutions of chitosan nanoparticles presented positive haemagglutination, without any change in reaction color. Chitosan nanoparticles presented hemolytic activity ranging from 186.20 to 223.12%, while neutralized solutions ranged from 2.56 to 72.54%, comparing to distilled water. Results highlight the need for development of new routes of synthesis of chitosan nanoparticles within human physiologic pH.

## 1. Introduction

Polymers, including chitosan, eventually cause haemagglutination [1–3]. Chitosan, mainly derived from chitin, is a polysaccharide made by randomly distributed chains of *β*-(1-4) D-glucosamine and N-acetyl-D-glucosamine. The formation process occurs by partial or total deacetylation of chitin on acid solution. The grade of acetylation must be lower than 20% and molecular weight around 200 kDa. The acetylated remaining portions on chains are responsible for compound solubility [4, 5].

Chitosan could be found on different forms, chain length, and grades of deacetylation. This diversity increases exponentially by chemical modifications which been evaluated [6]. One of these modifications is the ionic reticulated chitosan nanoparticle with specific polyanions. This gelification process occurs due to intra- and intercross links formation between chitosan diluted on acid solution and tripolyphosphate (TPP). These links occur between phosphate groups of TPP and amine groups of chitosan [7, 8].

Inert chitosan seems to be nontoxic biopolymer. The binding properties of this polymer to diverse surfaces increase its usefulness and potential application in medical sciences [9]. Recent work suggested that chitosan would be largely used in medical area demonstrated by its antioxidative, anti-inflammatory, anticancer, antimicrobial, and immunostimulatory and tissue repair inducing properties. Some chitosan-based peptides are also used as delivery systems for drugs, proteins, and genes [5, 10–13]. The lack of information about this potential application of chitosan nanoparticles in blood disorders in humans led us to perform the protocol presented in this paper. Also, according to previous data, all the components present in the whole blood alone and in combination with each other influence the hemostatic function of the blood [14]. Due to its cationic nature chitosan might be considered as a potential hemostatic agent. Once the amines are protonated by acidic pH, the positive charge is transferred to the protein chain. Since the majority of the biological membranes present anionic nature, the chitosan would be able to strongly adhere to them by electrostatic interactions. Chitosan-bound erythrocytes are believed to form a local net independently of the other hemostatic agents [5].

Hemagglutinating capacity of chitosan turned a promised hemostatic agent on reduced pre and postoperatory bleeding. The indication should be to elective and urged situations when the managing of bleeding is a challenge. Since pharmaceutical products might react with red blood cells and induce some undesirable reactions such as hemolysis, this must be addressed when evaluating the biocompatibility of any material intended for in vivo use. Acute effects of the hemolytic reactions are the impairment of the oxidative stress, induction of hypertensive peaks, and nitric oxide depletion, whereas chronic effects are dysregulated iron metabolism in the liver, atherosclerosis, and renal failure [15]. Various derived forms of chitosan are currently used to promote hemostasis on experimental studies to generate a compound with trading potential [1, 5]. The present work aimed to evaluate in vitro hemagglutination activity of chitosan nanoparticles using human erythrocytes and hemolysis effects of this formulation.

## 2. Material and Methods

### 2.1. Production of Chitosan Nanoparticles

The production of chitosan nanoparticles was made using inotropic gelification of chitosan solution with TPP suggested by Calvo et al. [16]. For this purpose, chitosan (≥75% deacetylation; product number: 448869; CAS number 9012-76-4, Sigma-Aldrich) was dissolved in hydrated glacial acetic acid solution (Química Moderna) diluted on 1% physiologic solution (solution 1) or distilled water (solution 2). The dissolution occurred over magnetic agitation during four hours at room temperature.

Adding, 0.2% of TPP was dropped (1000 *μ*L/min) into chitosan solution over magnetic agitation, staying for more 15 minutes after dropping process. The dispersions were diluted on 0.4; 1; 1.6; and 3 mg/mL, and part of this volume was neutralized by addition of 1 mol/L^−1^ NaOH. Dispersed nanoparticles on solution 1 were named solution A and dispersed on solution 2 and were named solution B. Solubilization of chitosan on solutions 1 and 2 was satisfactory and had an increased opalescence of the solution after TPP dropping over magnetic agitation, suggesting nanoparticle formation.

### 2.2. Transmission Electron Microscopy

**Figure 1:**
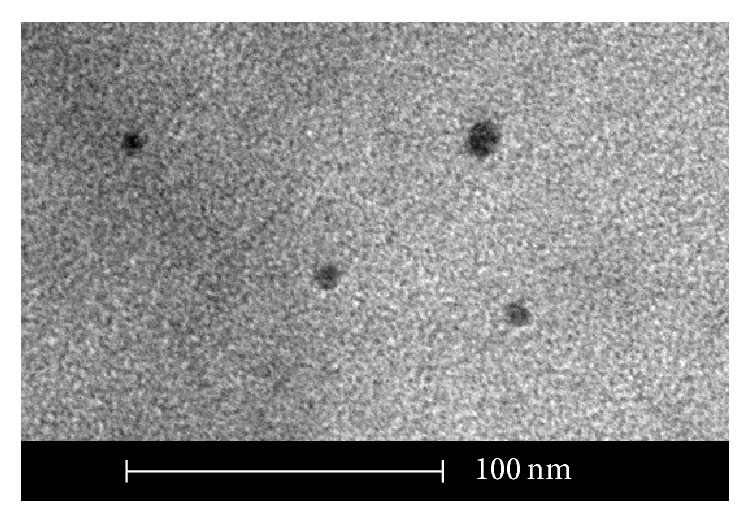
Transmission electron photomicrography showing chitosan nanoparticle structure in solution.

The sample was placed on a copper grid coated with formvar-carbon (Agar Scientific Ltd., Essex, UK), dried overnight in an oven at 27°C, and observed on transmission electron microscopy TecNai G2 Spirit TEM (FEI). The particles presented a medium size of 10 nm with a round shape (Figure 1).

### 2.3. Sterilization of Chitosan Nanoparticles

Chitosan nanoparticles were sterilized by direct exposition to UV radiation during 20 minutes. The effectiveness of this method was proved by direct microbiologic assays using BHI and Sabouraud Agar mediums to bacteria and fungi growth, respectively. The Petri dishes with chitosan nanoparticles were incubated on 37°C, for 24 h and 48 h, without any evidence of CFU formation for all tested samples.

### 2.4. Erythrocyte Sample Selection

Five healthy subjects, with 18 to 40 years of age, without hemoglobinopathies and without relatives on the first grade with these diseases were recruited. Peripheral blood samples were obtained by venopuncture on heparinized tubes (BD Vaccutainer).

After total blood collection, following a methodology described by Rao and Sharma [1], the samples were centrifuged to 1200–1500 rpm, during 15 minutes and plasma, platelets, and white cells were discharged. Erythrocytes were washed three times on 15 mL of buffer phosphate solution (pH 7.4).

### 2.5. Hemagglutination Assay

Hemagglutination assay was made according to Banerjee et al. [17] protocol. Erythrocytes were diluted on 2% phosphate buffer and 50 *μ*L of this dilution was transferred to “U” bottom 96-well microplates. The inoculation was made at room temperature. The proportion of erythrocytes and reagents was 1 : 1 (v : v) and performed in duplicate.

Solutions A and B were evaluated as test groups following the previous concentrations, with and without pH neutralization. Comparative groups also tested were 0.2% TPP, neutralized (1 mol/L^−1^ NaOH) and nonneutralized solutions 1 and 2, 1 mol/L^−1^ NaOH alone, saline solution (0.9% NaCl), phosphate buffer saline (PBS, pH 7.4), and distilled water.

Assay time was one hour and visual and photographic evaluation was made by a calibrated evaluator. Hemagglutination was described using a scale purposed by Stavitsky [18]:

 + + + + compact granular agglutinate;
 + + + smooth mat on bottom of tube with folded edges;
 + + smooth mat on bottom of tube, edges somewhat ragged;
 + narrow ring of red around edge of smooth mat;
 ± smaller area of tube covered than + and heavier ring around edge;
 − discrete red button in center of bottom of tube.

Other presentations were interpreted as undetermined event.

### 2.6. Hemolytic Activity

Hemolytic activity was performed following Singhal and Alok [19] method. One hour after addiction of reagents inside wells containing erythrocytes, 70 *μ*L of supernatant was transferred to flat bottom 96 well microplates to absorbance reading (GloMax-Multi) on 560 nm wavelength filter. Positive and negative controls were made by respective addiction of distilled water and physiologic solution to blood. The % of hemolysis was obtained using the following formula:

(1) $$\begin{array}{c} \%\text{Hemolysis}=\frac{\text{SA}-\;\text{NA}\;}{\;\text{PA}\;-\text{NA}}\times 100 \end{array}$$

as SA, PA, and NA corresponded to sample absorbance, positive absorbance, and negative absorbance, respectively. Hartmann et al. [20] index was used and a result lower than 5% was interpreted as acceptable for hemolysis.

## 3. Results

### 3.1. Hemagglutination Assay

As presented in Table 1, the visual determination of agglutinated erythrocytes was possible in all samples incubated with neutralized A and B solutions. Among these solutions, neutralized A and most of the neutralized B solutions presented complete hemagglutination index (+++), whereas 0.4 mg/mL neutralized B solution induced weak hemagglutination index (+). Moreover, the addition of PBS, saline, TPP, and 1 and 2 neutralized chitosan colloids did not present any evidence of hemagglutination.

Interestingly, the samples incubated with A, B, 1 and 2 solutions prepared under acid pH, and samples incubated with NaOH 1 mol/L^−1^ alone—without chitosan nanoparticles—presented a green-yellowish color. Distilled water induced the appearance of an opaque supernatant on incubated erythrocytes. The conditions of color change and medium opacity interfered the determination of the hemagglutination index of these samples.

### 3.2. Hemolytic Activity

After determing the hemagglutination index, the supernants were transfered to other 96-well plates and subjected to spectrophotometric analysis (560 nm wavelenght). As observed in Table 2, neutralized chitosan solutions presented lower hemolytic activity than chitosan nanoparticle solutions prepared under acid pH, at which supernatants showed elevated absorbance comparable to distilled water (hemolysis positive control).

## 4. Discussion

In this study, chitosan nanoparticle solutions were produced by ionotropic gelification protocol. The ionic interaction between 1% acetic acid solubilized chitosan and TPP dissociated in distilled water generates some polyanions such as OH^−^ and P_3_O^5−^_10_, the last being responsible for the binding with chitosan amino groups [21].

Synthesis of chitosan nanoparticles showed progressive opalescence after TPP dripping which suggests that small particles would be in the suspension [16]. Also, this change in opalescence presented by chistosan nanoparticles has been defined as being a typical Tyndall effect [22]. The mean size of 10 nm observed by TEM suggested that the nanoparticles produced here were within the range comprising 1 to 100 nm which denfines the nanoparticle scale according to the Standard Terminology Relating to Nanotechnology E 2456-06 [23]. The chitosan nanoparticles produced in this study were smaller than those produced by Mohammadpour Dounighi et al. [24], whose particles presented 150–350 nm. This observation indicates the need for implementing metrology standards related to chitosan nanoparticle suspensions.

The potential use of chitosan as an hemostatic agent has been demonstrated in literature. Previously, rabbit and human erythrocytes formed aggregates with disrupted shape when incubated with acetic acid solubilized chitosan particles, without the addition of TPP [25]. Other studies showed that 2% chitosan nanoparticle-based films also presented an agglutination effect in human erythrocytes [1], whereas polyphosphate-chitosan complex dressings demonstrated blood clotting in swine samples [2]. Moreover, it has been demonstrated that chitosan nanoparticles promoted agglutination of rabbit blood cells by binding to the erythrocyte membrane [3]. All these data have pointed to the hemagglutination ability of chitosan particles in solution or within nanostructured films, which agrees with the results presented here by the neutral pH chitosan nanoparticle solutions. In both saline and water diluted acetic acid the hemagglutination occurred, being the first even more intense in the Stavitsky and Jarchow scale [18]. Another aspect to be considered was the time of sample incubation with chitosan nanoparticle solutions in this study (up to 1 hour) and the period indicated in the literature mentioned above (varying from 2 to 20 minutes). Although it would sound to be an important issue, this variation of time should not be considered as a drawback for the comparison of the results obtained in these works by considering that hemagglutination assays are evaluated by visual analysis of each sample and that this would bring a subjectivity to the different protocols adopted in these studies [26]. Most importantly, however, all samples in this studied were analyzed within the same period of time.

All the erythrocytes incubated with A and B solutions, solutions 1 and 2 diluted in acid pH, and NaOH 1 mol/L^−1^ presented a brownish colour, distinct from the standard redish colour typically observed when samples were incubated with chitosan nanoparticles in neutral pH. This variation in the superanatant's colour impaired the visual examination of the samples and suggested the occurrence of an hemoglobin oxidative phenomenon, consequently an hemolytic event [27]. Hemolysis was assessed by spectrophotometric analysis of the supernatants in 560 nm wavelenght. All the samples incubated in acid pH solutions presented higher optical densities than neutralized solutions. The adoption of the 560 nm wavelenght encompasses the standard wavelength spectrum used to determine the occurrence of oxidative forms of human hemoglobin [28]. In order to evaluate a potential hemagglutination activity of any substance, keeping the natural reddish color of the sample is indispensable for a correct operationalization of the technique. Also the integrity of the hemoglobin structure might function as a key factor to determine the potential biocompatibility of nanoparticle solution [29]. The brownish color observed in the samples incubated with chitosan solutions prepared in acid pH also interfered with hemolytic evaluation, determining optical density values higher than those obtained with technique control (distilled water equivalent to 100% hemolysis).

All tested solutions presented elevated hemolytic activity, except in neutralized B chitosan solution 0.4 mg/mL, at which hemolytic activity was calculated to be 2.56% of the values of samples incubated with H_2_O_distilled_. This percentage of hemolysis would be considered insignificant to cause any side effect in humans [20]. When comparing the absorbance values between neutralized A and neutralized B solutions, it would be evidenced that the use of saline (NaCl 0.9%) in A solution as an alternative acetic acid diluter increased its compatibility to erythrocyte integrity than commonly used distilled water.

The other important issue to be discussed is that pH itself interferes with hemagglutination process by decreasing erythrocyte binding forces. Generally, better agglutination results were found when erythrocytes were incubated with solutions which pH ranged from 6.4 to 8.4—close to the physiological pH [29]. Despite the possible induction of structural modifications in chitosan nanoparticles, the oxidative effect on human erythrocytes promoted by the acetic acid present in the chitosan solution was minimized by its neutralization with a strong base. Although large variations on the pH of some solutions used for nanoparticle synthesis might promote alterations in size, shape, and dispersion rate of the polymers, it was demonstrated that only above pH 8.0 a lack of TPP crosslinking with chitosan structured would decrease nanoparticle viability [9].

Moreover, the deacetylation degree of chitosan particles must be considered when evaluating the physicochemical and biological properties of this polymer. The majority of the commercial chitosan polymers have average degree of deacetylation of 70–90% [30]. Higher DDA are believed to be better for biological applications of chitosan, but additional deacetylation steps would increase the total cost of the product and might result in a partial loss of polymer crosslinking, impairing the viability of the chitosan micro- and nanoparticles [30–32]. Our idea is that the interaction between chitosan nanoparticles and erythrocytes forms a net which might be responsible for the erythrocyte grouping. Because of that, one would consider that polymer stability is fundamental for the biological function of the chitosan nanoparticles even producing them with lower deacetylation degrees.

The strategy of simply acid neutralization by addition of a strong base would not be the ideal choice for chitosan nanoparticle production for human use. A more controlled strategy might be applied to maintain chitosan nanoparticle stability and human biocompatibility. Authors have adopted the ultracentrifugation technique to diminish the residual amount of acetic acid and other solvents in shrimp-derived chitosan nanoparticle synthesis [33]. Although efficient for supernatant removal, the chitosan nanoparticles presented aggregate formation and lost their nanostructured organization. Higher dispersion rates of nanoparticles were associated with worse functionality and interaction with target cell membrane [29].

## 5. Conclusion

The ionotropic gelification method was efficient and affordable enough for the synthesis of large volumes of chitosan nanoparticle solutions. The pH neutralization of these solutions induced higher hemagglutination index and lower hemolytic activity in human erythrocytes than chitosan nanoparticles conventionally synthetized under acid pH. Noteworthy, the development of new routes for the production of large volumes of chitosan nanoparticles is needed to achieve clinical applications of these solutions in a neutral pH compatible with human blood cells and tissues.

## Figures and Tables

**Table 1:** Percentage of hemagglutination reactivity of human erythrocytes incubated with chitosan nanoparticles.

Solution [mg/mL]	Hemagglutination (%)^*^					
	++++	+++	++	+	±	Indeterminate^**^
	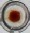	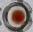	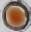	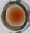	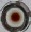	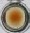

Solution A [0.4]	—	—	—	—	—	100
Solution A [1.0]	—	—	—	—	—	100
Solution A [1.6]	—	—	—	—	—	100
Solution A [3.0]	—	—	—	—	—	100
Solution A [0.4]^***^	100	—	—	—	—	—
Solution A [1.0]^***^	100	—	—	—	—	—
Solution A [1.6]^***^	100	—	—	—	—	—
Solution A [3.0]^***^	100	—	—	—	—	—
Solution B [0.4]	—	—	—	—	—	100
Solution B [1.0]	—	—	—	—	—	100
Solution B [1.6]	—	—	—	—	—	100
Solution B [3.0]	—	—	—	—	—	100
Solution B [0.4]^***^	—	—	—	100	—	—
Solution B [1.0]^***^	—	80	—	—	20	—
Solution B [1.6]^***^	—	90	10	—	—	—
Solution B [3.0]^***^	—	100	—	—	—	—

^*^Percentage of the wells compatible with Stavitsky (1954) [18].

^**^Indicates the percentage of the samples that showed color modification that distorted the visual definition of the hemagglutinating event.

^***^Neutralized solution by the addition of NaOH 1 mol/L^−1^.

**Table 2:** Hemolytic activity of the diverse chitosan nanoparticle formulations in human erythrocytes.

Solutions	Optical density 560 nm	Hemolysis %
Distilled water	0.137 ± 0.032	+control
NaCl 0.9% saline solution	0.034 ± 0.009	−control
Solution A [0.4 mg/mL]	0.226 ± 0.024	186.20
Solution A [1 mg/mL]	0.237 ± 0.014	197.16
Solution A [1.6 mg/mL]	0.234 ± 0.021	194.07
Solution A [3 mg/mL]	0.252 ± 0.026	212.02
Solution A neutralized [0.4 mg/mL]	0.037 ± 0.004	2.56
Solution A neutralized [1 mg/mL]	0.047 ± 0.018	12.97
Solution A neutralized [1.6 mg/mL]	0.045 ± 0.007	10.44
Solution A neutralized [3 mg/mL]	0.059 ± 0.011	23.95
Solution B [0.4 mg/mL]	0.228 ± 0.018	188.72
Solution B [1 mg/mL]	0.234 ± 0.018	193.91
Solution B [1.6 mg/mL]	0.238 ± 0.020	198.46
Solution B [3 mg/mL]	0.264 ± 0.027	223.12
Solution B neutralized [0.4 mg/mL]	0.088 ± 0.124	52.48
Solution B neutralized [1 mg/mL]	0.052 ± 0.010	17.56
Solution B neutralized [1.6 mg/mL]	0.109 ± 0.109	72.54
Solution B neutralized [3 mg/mL]	0.063 ± 0.010	28.19
